# Poly(ADP-ribose) in Condensates: The PARtnership of Phase Separation and Site-Specific Interactions

**DOI:** 10.3390/ijms232214075

**Published:** 2022-11-15

**Authors:** Elizaveta E. Alemasova, Olga I. Lavrik

**Affiliations:** 1 Institute of Chemical Biology and Fundamental Medicine, SB RAS, Novosibirsk 630090, Russia; 2 Department of Natural Sciences, Novosibirsk State University, Novosibirsk 630090, Russia

**Keywords:** liquid–liquid phase separation (LLPS), poly(ADP-ribose) (PAR), PARP, condensate

## Abstract

Biomolecular condensates are nonmembrane cellular compartments whose formation in many cases involves phase separation (PS). Despite much research interest in this mechanism of macromolecular self-organization, the concept of PS as applied to a live cell faces certain challenges. In this review, we discuss a basic model of PS and the role of site-specific interactions and percolation in cellular PS-related events. Using a multivalent poly(ADP-ribose) molecule as an example, which has high PS-driving potential due to its structural features, we consider how site-specific interactions and network formation are involved in the formation of phase-separated cellular condensates.

## 1. Introduction

*Liquid–liquid phase separation (LLPS)* is a process of demixing of a homogeneous polymer solution into two phases containing different concentrations of macromolecules. According to the basic LLPS model, the selective concentration of desired molecules can occur spontaneously because of the laws of thermodynamics.

Nonetheless, in order for phase separation (PS) to occur without changing physical parameters (e.g., temperature and volume, which is usually not the case for the formation of condensates in the cell), the system must approach a threshold concentration (*saturation concentration*, $C_{sat}$), which can hardly be achieved due to nonspecific interactions, especially under conditions of strong competition in cellular plasma. Therefore, it has been assumed that so-called *restricted* PS can be implemented in the cell, that is, PS initiated by highly specific heterotypic site-specific interactions (SSIs) that function under a one-phase regime to achieve $C_{sat}$ [1].

A link between the LLPS concept and the classic paradigm of SSIs in molecular organization is also evidenced by the fact that many polymers can undergo *percolation*: a networking transition allowed by the multivalence of sequence-, chemistry-, and structure-specific interactions [2].

Poly(ADP-ribose) (PAR) is a nucleic-acid-like polymer (polyA) with a highly heterogenous, low-complexity, flexible structure. PAR is highly charged, has distinctive structural elements, and may be in the form of a site-specific post-translational modification (PTM). It participates in the organization of numerous cellular condensates and PS-related processes. In this review, we discuss how certain PAR structural features determine its ability to act as a driver of site-specific, nonspecific, and network-forming interactions. We also look at several examples of phase-separated cellular condensates that involve PAR to understand how SSIs and networking participate in their assembly.

## 2. PS: A Basic Model

Biological polymers undergo LLPS due to the action of two synergistic forces: intermolecular interactions and H_2_O entropy [3]. To put it simply, a polymer solution undergoes LLPS in the range of polymer concentrations in which the graph of Gibbs free energy change on mixing ($\Delta G_{M}$) (plotted for this system) acquires negative curvature. LLPS may be considered a density transition [2].

### 2.1. Physics of LLPS. Part 1: Intermolecular Interactions

The thermodynamics of a phase equilibrium in polymer solutions is based on Flory–Huggins theory [4,5]. When a polymer is mixed with a solvent, it disperses in the solvent and behaves as if it were also a liquid [6]. The fundamental thermodynamic equation used to describe such systems relates Gibbs free energy (*G*) to enthalpy (*H*) and entropy (*S*): G=H−TS, where *T* is temperature [6]. In Flory–Huggins theory, the entropic and enthalpic contributions to the Gibbs free energy are calculated separately, using the lattice model. The assumptions made by Flory–Huggins theory can be found in books [6,7]). Entropy is calculated as the configurational entropy of mixing a polymer with solvent molecules, while an enthalpy change upon mixing is assumed to arise from the formation of new solvent–polymer contacts, replacing some contacts present in pure components before the mixing [6]. The calculation involves some algebra detailed in ref. [6]. For polymers, the contribution of entropic term $-T\Delta S_{M}$ to $\Delta G_{M}$ is small due to the low probability of molecular rearrangements, and the enthalpic contribution plays a primary role [8]. The interaction of molecules in solution is characterized by Flory–Huggins parameter χ. When χ>0, the entropic contribution under definite conditions becomes insufficient for the $\Delta G_{M}$ function to remain in the range of negative values, and PS in this case becomes thermodynamically more favorable. The *saturation concentration* ($C_{sat}$) of a given LLPS-driving macromolecule is defined as the equilibrium concentration above which the homogeneous solution becomes phase-separated [9]. Basic principles of PS have been extensively described [10,11,12,13,14].

### 2.2. Physics of LLPS. Part 2: H_2_O Entropy

The role of synergistic action of H_2_O entropy in LLPS is unclear. Initially, it was proposed that because the molecules of water bound to the surface of macromolecules are more ordered, their release from the surface into the solution increases solvent entropy [3,15].

Recently, by a combination of terahertz spectroscopy and fluorescence microscopy, it was confirmed that the release of a preordered hydration shell water into the bulk is the actual thermodynamic driving force behind LLPS [16]. The key concept is the existence of distinct populations of water molecules hydrating polar and hydrophobic patches of a protein [16]. Within this paradigm, LLPS is promoted by the greater tetrahedral coordination of water molecules (bound water) and by the minimization of less favorable interactions of water molecules (wrap water) related to hydrophobic patches, similarly to protein folding [16].

## 3. SSIs and the Concept of *Restricted* PS

Despite the simplicity and elegance of the LLPS hypothesis, there are certain doubts that *general* PS driven by nonspecific interactions of macromolecules as associative polymers can seed the formation of membraneless compartments under physiological conditions. Thus, it is unclear how the low specificity can result in the selective concentration of desired biomolecules in the presence of thousands of competing interactions. It is also unclear how a limited set of weak nonspecific interactions can regulate the fusion/immiscibility of individual phase-separated droplets [1].

Indeed, it has been proposed that the incompatibility of liquid phases is ensured by specific molecular interactions [17], and multiphase immiscibility within condensates can be achieved by the modulation of relative protein–oligonucleotide binding affinity levels [18]. For example, in experiments with domain truncations, it has been found that the mutual exclusion of fibrillarin (FIB1)- and nucleophosmin (NPM1)-containing liquid phases of the nucleolus is encoded not in intrinsically disordered protein regions (IDRs) but in these proteins’ RNA recognition motifs, which have different RNA substrate specificity [17]. The involvement of RNA-binding domains in the maintenance of multilayer organization has also been shown for stress granules (SGs). For instance, the removal of the RNA-binding domain of the key SG protein, G3BP, rather than its specific region responsible for the interaction with other proteins, and this leads to the fusion of previously incompatible phases in reconstructed SGs [19]. Additionally, immiscibility is dictated by the difference in surface tension between different condensates [20]. Specific interactions between RNA and RNA-binding domains in turn are critical for the regulation of surface tension of various phases [21,22,23,24].

According to the mechanism proposed by Musacchio [1], the assembly of nonmembrane compartments begins with highly specific heterotypic SSIs that occur at concentrations below $C_{sat}$ of any interacting component, i.e., under the one-phase regime. Nevertheless, low-specificity interactions of PS drivers at concentrations above $C_{sat}$ can result in liquid demixing of cellular plasma, implementing the concentration of additional molecules and the formation of certain material properties of a mature condensate (so-called *special* or *restricted* PS) [1]. A synergy between stoichiometric SSI and stochastic binding was recently confirmed by a combined theoretical-experimental analysis of a complex coacervate organized by SynGAP and PSD-95 proteins [25]. Of note, while in the dilute phase, these two proteins form a homogenous 3:2 complex, and the phase behavior of SynGAP–PSD-95 inferred from experimental data is inconsistent with stoichiometric-complex-driven LLPS and reveals the involvement of nonstoichiometric auxiliary interactions in the assembly of SynGAP–PSD-95 coacervates [25].

Actually, compartments usually form around certain spatial and temporal signals that attract and concentrate desired molecules at a target locus. If the concentration of self-interacting molecules is below supersaturation, the formation of separate phases is energetically unfavorable, and molecules will bind to nucleation sites giving rise to small clusters but not phase-separated droplets [26]. If a phase-separated droplet is nucleated by specific signals, it should persist after their termination [1,26]. Such examples can also be found at least in vitro (FUS and PAR, see below), but most cellular phase-separated condensates exist only during their formative SSIs (accordingly, the maintenance of nucleoli requires the continuous active synthesis of ribosomal RNA (rRNA) via transcription [27]).

Oligonucleotides act as ultra-high-valency molecules (“superscaffolds”) that increase molecular connectivity among scaffold proteins [18]. By means of a coarse-grained model, it has been found that they can facilitate protein LLPS via a seeding-type mechanism: by recruiting multiple protein molecules and decreasing the kinetic barrier for nucleation [18]. In vivo, such a mechanism is realized by long noncoding RNAs (lncRNAs), for example, by lncRNA NORAD during the regulation of Pumilio proteins or by lncRNA Xist in the process of X-chromosome inactivation/dosage compensation [28]. In both cases, the condensate is nucleated by stoichiometric multivalent interactions of the lncRNA with its protein partner, thereby causing a local increase in protein concentration. At the second stage, the supra-stoichiometric recruitment of additional protein molecules is mediated by homotypic interactions between protein IDRs [28].

## 4. Polymer Networks and the Concept of *Percolation-Coupled* PS

A bridge between the LLPS concept and the classic structure–function paradigm is revealed by the fact that multivalent macromolecules with sticker-and-spacer architectures also undergo *percolation*, forming a network of reversible associations [2]. These physical crosslinks are enabled by specific interaction motifs (*stickers*) that can consist of folded domains, IDRs, or even individual residues. *Spacers* or linker sequences influence solubility and therefore modulate the extent of coupling between PS and percolation [2,29]. A detailed discussion of the sticker-and-spacer framework in the context of condensates can be found in ref. [30].

Intersticker crosslinks result in the appearance of molecular clusters with distinct stoichiometries. If there are more than three stickers per molecule, an association of stickers occurs not intramolecularly but mainly between different molecules [30,31]. The *percolation threshold ($C_{perc}$)* is the concentration above which a larger cluster becomes system-spanning. Below $C_{perc}$, the system is composed of organized clusters of finite sizes and stoichiometries, whereas above $C_{perc}$, all molecules are incorporated into the percolated network [2].

According to the classic Flory–Huggins theory, clusters larger than three to five molecules do not form below $C_{sat}$ [29]. Nonetheless, the formation of clusters in subsaturated solutions and of phase-separated condensates in supersaturated solutions has been confirmed experimentally for RNA-binding proteins containing RNA-binding and low-complexity domains [29]. Mutations in sticker regions reduce the formation of clusters and raise $C_{sat}$ for PS, indicating a strong coupling between both processes [29]. It has also been clearly demonstrated that the overall phase behavior of FUS-like proteins is governed by distinct, sequence-specific energy scales, with cluster formation and PS being decoupled by solutes that affect protein solubility or by specific types of mutations [29].

**Figure 1:**
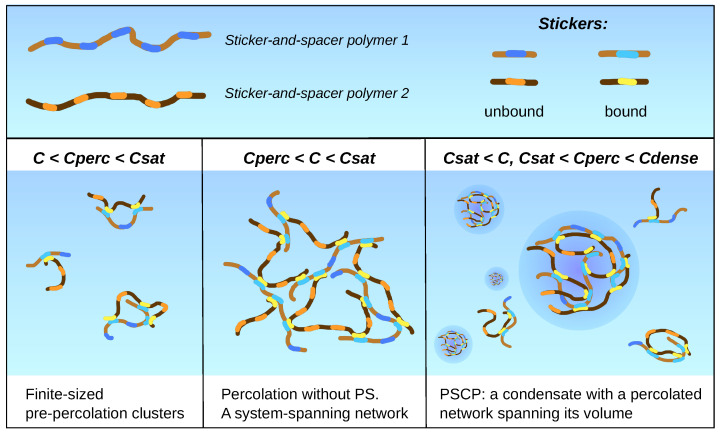
The relation between phase separation (PS) and percolation.

Although PS is a density transition triggered by the sum total of interactions contributing to χ, percolation is a networking transition allowed by the multivalence of sequence-, chemistry-, and structure-specific interactions [2]. In this regard, the phase behavior of associative polymers is realized by renormalized interaction parameter $\chi^{\prime }=\chi +\Delta \chi$, where Δχ corresponds to contributions of specific interactions [32]. The specific binding of macromolecules mediates networking above $C_{perc}$, and the relation between $C_{sat}$ and $C_{perc}$ determines whether PS and percolation are coupled or decoupled in a given system [33] (Figure 1). Condensates that assemble via *PS coupled to percolation (PSCP)* are permeated by a network of specific contacts and behave as viscoelastic network fluids [2]. In their recent work, T. Mittag and R. V. Pappu discuss how the PSCP notion can help answer many challenging questions about the applicability of the PS paradigm to the live cell [1,34].

## 5. PAR and Its PS-Driving Potential

### 5.1. PTMs by Polymers

Interactions are considered to be the main reason for PS regulation. PTMs affect different types of interactions, including covalent and noncovalent bonds, electrostatic interactions, and hydrophobic and hydrophilic forces and therefore represent one of the key ways for regulating intracellular PS events [35].

Among more than 200 PTMs described to date, especially interesting in the context of PS are modifications by long polymeric chains having a complex heterogeneous structure. Two key examples are the addition of polypeptides such as ubiquitin (ubiquitination) or ubiquitin-like modifiers (in particular, SUMOylation) [36] and nucleic-acid–like polymers (PARylation).

Ubiquitination regulates an assortment of processes in eukaryotic cells owing to its propensity to form various structures [37]. Ubiquitin (Ub) can be covalently attached to modification targets as a monomer on one or more sites or can form chains where Ub units are joined via isopeptide bonds. PolyUb chains may be homotypic, where monomers are linked uniformly through the same acceptor site of Ub, and heterotypic, which contain multiple types of linkages. Ub subunits in “mixed” heterotypic chains are modified on a single acceptor site, whereas “branched” polyUb chains contain ubiquitins modified on multiple acceptor sites [37]. There is evidence that the structural and conformational properties of polyUb chains can contribute to PS with ubiquitin-binding effectors [38]. By facilitating or inhibiting PS, polyUb chains may influence signaling outcomes in the cell, similarly to PARylation (see below): another PTM, which can also regulate PS via length and branching [38,39]. Small Ub-like modifiers (SUMOs) can be attached to protein targets too as one or multiple monomers or in the form of various types of polymer chains, including branched ones. Despite the similarities between SUMO and Ub, there are some differences (for example, the surface charge distribution), which endow SUMO with unique cellular functions [40]. PolySUMO chains are recognized by noncovalent binding with SUMO-interacting motifs. The latest evidence supports the important role of SUMO in the assembly and disassembly of condensates [41].

In the present review, we focus on PARylation and first of all discuss in detail the structural features of PAR that determine its ability to contribute to site-specific, nonspecific, and network-forming interactions.

### 5.2. PAR

This is a nucleic-acid-like polymer synthesized from NAD${}^{+}$ by ADP-ribosyltransferases (ARTs) capable of performing the elongation. In mammals, only four enzymes from the 17-member ART protein family (PARPs 1 and 2 and tankyrases 1 and 2) can generate PAR [42,43]. An updated nomenclature of mammalian ARTs is proposed in another review [44].

During ADP-ribosylation, ADP-ribose residues are transferred to a modification target, which can be a protein, DNA [45,46,47], or RNA molecule [48]. It was shown recently that mammalian DNA is physiologically PARylated to various levels in tissues [49]. Noncanonical ADP-ribosylation reactions are discussed in ref. [50].

The length of PAR chains produced by PARP1—the central cellular PAR polymerase [51]—may exceed 100 nm [52,53]. As a result of automodification, PARP1 is surrounded by a halo of PAR strands that can branch and reach 200 monomers and more [54,55]. How such an intricate structure can be formed is not entirely clear [56].

To become catalytically active, PARP1 [57] and PARP2 [58] should undergo conformational changes that relieve enzyme autoinhibition. These structural rearrangements are triggered by PARP1’s or PARP2’s interaction with DNA strand breaks, which therefore are the main signal and epicenter of PAR synthesis. Nevertheless, there is evidence that PARP1 and -2 may activate in other ways: through interaction with non-B DNA [59], RNA [60,61,62] (refuted by [63]), including small nucleolar RNAs [64], and even PAR itself [65]. The substantial overactivation of PARP1 induced by the addition of free PAR in the presence of effector protein YB-1 has been demonstrated as well [66].

Of note, it is reported that the multiple domains of PARP1 can cooperate in response to interactions with different PARP1 targets, thereby leading either to short-term or to long-lasting activation of the enzyme [67]. Although PARP1 association with damaged DNA mediated by its DNA-binding domain triggers short-term activation, the interaction of the PARP1 C-terminal domain with histone H4 induces prolonged PARP1 activity [67]. Recent evidence for long-lasting PARP1 activation by various signal transduction mechanisms (under physiological conditions) interfering with DNA-dependent activation is discussed by Cohen-Armon [68]. Tankyrases do not contain the autoinhibitory domain that limits their catalytic activity [69] and therefore can also generate PAR regardless of the presence of a DNA lesion. Moreover, within the cell, PAR can be translocated in the form either noncovalently bound to proteins or covalently attached to modified targets. Endogenous PAR can be released into the extracellular space [70]. Extracellular PAR can also appear because of the activity of PARP2 located on the surface of T cells [71]. Thus, although up to 90% of cellular PAR is synthesized by PARP1 near DNA strand breaks [72], this polymer can be found almost everywhere inside and outside the cell and participates in the formation of nonmembrane structures.

PAR is degraded mainly by PAR glycohydrolase (PARG), which possesses both exo- and endoglycosidase activities [73]. Short PAR chains are slowly processed by PARG [74], and this process can be compensated by (ADP-ribosyl)hydrolase 3 (ARH3) activity. The latter enzyme also hydrolyzes terminal seryl-ADP-ribosyl linkages [75], which are inaccessible to PARG [76]. PARG, but not ARH3, can resolve branched point PAR architecture [77].

### 5.3. PAR as a Driver of Site-Specific, Nonspecific, and Network-Forming Interactions

Multivalent interactions, the presence of flexible molecules, and electrostatic binding between highly charged molecules are the key factors promoting LLPS [78]. All these features are inherent in the structure of PAR, giving this polymer a strong ability to directly participate in PS. Moreover, the low complexity and polyA sequence of PAR may predispose this polymer to PS and to the formation of intermolecular interaction networks. Due to the presence of specific structural elements and variations in polymer length and branching as well as to the specificity of modification sites in PARylated proteins, PAR can be also take part in SSIs.

#### 5.3.1. Specific Recognition of PAR

Different structural elements of a PAR molecule (iso-ADP-ribose, ADP-ribose–ADP-ribose junction, and terminal ADP-ribose) can be recognized and bound by an assortment of protein domains. These PAR reader modules include PAR-binding motifs, macrodomains, PAR-binding zinc fingers, WWE domains, and other polypeptides ranging from completely folded PAR-binding domains to disordered sequence stretches [79]. PAR molecules are very heterogeneous in terms of chain length and branching. These characteristics influence the set of PAR readers able to interact with a single PAR molecule and therefore determine their local concentrations. A polymer with a highly branched shape may limit the space available for PAR-binding domains of proteins that recognize linear PAR motifs [39].

Nevertheless, as we will see below in examples of PAR-involving cellular condensates (section “PAR and PS in the cell”), the “PAR code” of the polymer length and branching pattern, which regulates specific PAR–protein interactions [80,81], is rarely used for PS initiation. (Possibly, SSIs of transcription factors with PAR can play a scaffolding role during the assembly of the nucleolus and transcription hubs.) More likely, the specific recognition of the PAR structure is used not for triggering the PS process but for the subsequent recruitment of certain clients.

#### 5.3.2. PAR as a PTM

PTMs can change the charge, conformation, nucleic-acid binding, and other properties of proteins important for their PS behavior [82]. For instance, tau protein phosphorylation effectively enhances the kinetics of tau LLPS (a nonphosphorylatable tau mutant is unable to form droplets) [83]. A PTM can also alter the conformation and therefore the interaction profile of the modified protein [84] and regulate protein and RNA complex coacervation [85,86].

As a PTM, PAR participates in protein PS related to targeting to SGs [87,88,89,90]. In this case, however, PAR also does not perform a compartment-forming function because the targeted proteins discussed in such studies cannot be considered scaffolds during SG assembly (unlike G3BP proteins, without which SGs fail to form [91]).

It can be assumed that, as a PTM, PAR also participates more in attracting clients than in the formation of phase-separated compartments except for PARP1 automodification, which should be more properly regarded as the site of polymer synthesis, which serves as a specific signal for PS.

#### 5.3.3. PAR as a Multivalent Platform for a Substoichiometric Mechanism of PS Seeding

Either in the form of a PTM or in a free state, PAR has a surface composed of repeated ADP-ribose units, which can act as a multivalent platform for protein recruitment. Greater PAR valence can be achieved either by increasing the number of modification sites on a given protein or by increasing PAR length and/or branching [92]. In the case of lncRNAs, their repetitive sequences are essential for substoichiometric seeding of phase-separated condensates [28,93].

A recent work offers an interesting example of how PAR repetitiveness can be used for the functional clusterization of a PAR-interacting protein. It was found there that simultaneous PAR binding by several PARP13 molecules serves for the organization of enzyme clusters with manifold recognition sites for the binding of multiple CpG dinucleotides in viral RNA necessary for its degradation [94].

#### 5.3.4. Low Complexity and polyA Structure

The proteins known as LLPS drivers usually contain low-complexity domains or IDRs enabling weak and multivalent interactions for LLPS. Unstructured sequences in nucleic acids may play a similar part. In the cell, stress-induced low-complexity ribosomal intergenic RNA drives the formation of nucleolar liquid-like foci during nucleolus conversion to an A-body [95]. It has also been proposed that SGs are RNA analogs of unfolded protein aggregates and form at least partially via intermolecular RNA–RNA interactions due to exposed RNA surfaces [96]. mRNAs that fold into conformations with more exposed single-stranded-RNA are reported to partition more efficiently into ribonucleoprotein granules as compared to RNAs with less exposed single-stranded stretches [96].

Almost any two RNAs can interact, especially at high concentrations and if they do not fold into a strong self-structures [97]. mRNAs carrying large disordered regions are prone to form an extensive intermolecular interaction network, which drives the assembly of condensates with mesh-like shapes [98]. The unstructuredness of RNA correlates with the ability to adopt diverse structural conformations. The prediction of RNA fold-based structures has revealed that sphere-forming RNAs have stronger secondary structures than do network-forming RNAs [98]. Indeed, the addition of strong local base-pairing causes an RNA molecule to lose its capacity for network formation. Nevertheless, the predominantly structured RNAs that are able to engage in pervasive intermolecular RNA–RNA interactions (for example, RNA dimerization motifs) can also give rise to mesh-like condensates [98].

By means of a coarse-grained model, it was shown recently that trinucleotide repeats of RNAs when being recruited inside liquid droplets undergo transition from a hairpin-like conformation to an extended state, thus forming an extensive intermolecular network, which constrains the RNA conformational fluctuation and mobility [99].

In the case of RNA, the random coiled state of the molecule can offer greater accessibility to the binding of short cationic molecules than structured RNA can [100]. Complex coacervates composed of low-complexity RNA (polyU) and short polyamines are similar to the coacervates formed by IDR-containing peptides [100]. All four RNA homopolymers have also been found to self-assemble in the absence of polycations [101]. In particular, polyA forms asymmetrical assemblies with very slow relaxation rates [101]. It is noteworthy that the droplet formation ability of adenine is the highest among DNA nucleobases [102]. For instance, among 5-nt homo-oligomeric DNAs, only the DNA containing adenine gives rise to droplets in the presence of a polycation [102].

A recent work highlights the key role of an RNA component in the maturation of RNA–protein condensates. It was demonstrated there that RNA alone forms a viscoelastic network, the properties of which may be tuned by changes in the ATP: ${Mg}^{2+}$ ratio or temperature. In contrast, the protein component is free to diffuse [103]. Notably, polyA oligoribonucleotides form a stronger network compared to polyU, because model NPM1–polyA condensates undergo dynamic arrest at high ${Mg}^{2+}$ concentrations, whereas NPM1–polyU droplets remain liquid-like [103].

#### 5.3.5. Increased Flexibility

During LLPS, nucleic-acid chains are bent to achieve a conformation optimal for maximal charge neutralization [104]. Therefore, more rigid DNA molecules form weaker condensates that dissolve at lower ionic strength as compared with DNAs of equal length and charge density but with higher flexibility [104].

PAR has higher flexibility compared with single-stranded DNA or RNA, which is likely due to the presence of two phosphates linking the two sugar moieties instead of one as in DNA or RNA [105]. Its structural flexibility in solution has been explored by molecular dynamics simulations. It has been found that PAR polymers, especially long ones, adopt numerous different conformations in water and never reach a stable and defined 3D structure. A PAR chain consisting of 25 ADP-ribose units assumes a three-globe-shaped conformation, indicating a tendency to form a dynamic size- and branching-dependent multiglobular fold [105]. This intrinsic flexibility may allow PAR to recognize multiple proteins and specifically fit the structure of interaction partners [105].

It is reported that RNA secondary structure can regulate RNA sorting into distinct droplets by altering the ability to form intermolecular (RNA–RNA and RNA–protein) interactions [106]. PAR does not have a secondary structure, which may be related to observations that in contrast to RNA, there is no in vivo or in vitro evidence of the coexistence of immiscible PAR-organized phases (despite the notion of “PAR code” [80,81]). Nonetheless, it is possible that in the form of a PTM, PAR may possess more specifically organized structure than free PAR chains do [107,108].

#### 5.3.6. Charge Density and Distribution

Compared to canonical nucleic acids, PAR has a twice higher negative charge and additional space between ribose residues. It is the most electronegative natural polymer [109]. Charge density and distribution are key to complex coacervation [110]. A recent article provides extensive evidence that PS can be based on the central principle of charge complementarity that does not require flexible polymers, multivalency, specific interaction sites, disorder, or specific secondary structures [78].

On the other hand, polyelectrolyte interactions can drive the organization of ultra-high-affinity binding complexes with extreme disorder and highly dynamic behavior [111,112].

## 6. PAR and PS in the Cell

PAR is involved in the organization of numerous natural condensates whose assembly is linked with PS—the nucleolus [113,114], transcription hubs [115], DNA repair foci [116,117,118], SGs [88,89,119], ASK3 condensates [120], and the spindle [121]—and participates in PS-dependent processes such as biomineralization [70,122] and pathological protein aggregation [123,124]. Nonetheless, the exact role played by PAR in these events is still unclear and needs further investigation. In this section, we will try correlating the role of PAR with the possible type of PS-driving interactions using relevant examples of PAR-involving condensates or PS-dependent processes from the literature. In many cases, contributions of different types of interactions are very difficult to distinguish; hence, we will propose a putative arrangement transitioning from SSI-dependent (restricted) PS through networking to general PS.

### 6.1. PAR and SSIs

#### 6.1.1. DNA Repair Foci

DNA lesions initiate the formation of temporary compartments that concentrate damaged DNA and the repair machinery. PAR synthesis by activated PARP1 is the earliest event in the DNA damage response (DDR) because PAR levels peak at 1 min after a genotoxic insult [125]. It has been demonstrated in vivo that PAR can seed the LLPS of FET proteins, in particular, FUS, at lesion sites [116,117]. Atomic force microscopy studies offer an in vitro model for the formation of DNA repair foci: FUS binding to PAR arising at a DNA strand break site results in the assembly of compartments that accumulate PAR, FUS, and damaged DNA molecules [118]. It has been found that FUS-dependent LLPS is necessary for the initiation of the DDR, and LLPS inhibitors and LLPS-deficient FUS mutants impair the recruitment of DDR factors and the proper arrangement of γH2AX foci [126].

Positively charged RGG modules of FUS are necessary for its interaction with PAR [116], while a disordered low-complexity domain of this protein is crucial for the compartment formation [118]. The results obtained by Kar and coauthors have revealed PSCP for FUS-like proteins and RNA [29]. Nonetheless, for the FUS PS induced by PAR, in contrast to RNA-induced FUS PS, there are nuances, and therefore, we will consider this example later (in the section “Pathological aggregates”). Here, it is important to highlight to the following: the primary link in the chain of events of DNA repair foci formation is clearly a typical SSI: the interaction of PARP1 (PARP2) with damaged DNA. It is this interaction that is necessary for place- and time-specific PAR synthesis and for subsequent restricted PS of FUS causing the assembly of the repair compartment.

#### 6.1.2. The Nucleolus

The nucleolus represents a multilayered condensate that plays a fundamental role in ribosome biogenesis. In mammalian cells, the internal architecture of nucleoli consists of three nested subcompartments: the fibrillar center (FC), the dense fibrillar component (DFC), and the granular component (GC). rRNA transcripts are synthesized at the FC–DFC interface and are bound by FIB1, which is a prominent protein within the DFC. When passing through the DFC, nascent transcripts undergo initial processing and modification before proceeding into the GC layer enriched in the NPM1 protein, for the further maturation of ribosomal subunits [27]. Most eukaryotes, including budding yeast, have two nucleolar layers with a single fibrillar component [127]. The evolutionary divergence of this ancient FC into the modern FC and DFC was due to the extensive expansion of ribosomal DNA (rDNA) intergenic spacers [127].

The key significance of PAR for nucleolus organization is supported by the finding that up to 50% of PARP1 and PAR are localized to the nucleolus and that an impairment of PARP1 enzymatic activity or overproduction of PARG causes nucleolar disintegration in *Drosophila* [113]. This nucleolar fragmentation can be detected by an anti-FIB1 antibody, and the fragments do not contain rDNA. In contrast, the mutation of PARG results in nucleoli with heavily condensed regions. In the absence of functional PARylation, a defect in rRNA processing and a substantial reduction in ribosome assembly are observed; it has been proposed that PARP1 activation occurs simultaneously with the transcriptional start by Pol I and the generated PAR targets nucleolar proteins to precursor rRNA [113]. It is thought that PAR may have played a role in the evolutionary FC–DFC division [114] because there is no PARylation in yeast [128] even though they have a long rDNA spacer between 35S RNA coding sequences [129]. From the results obtained on *Drosophila* nucleoli, it can be concluded that PAR is concentrated in the DFC, where it forms a network of functional interactions with rRNA and FIB1 [113]. Indeed, as we mentioned above, multiphase immiscibility within condensates can be regulated by protein–oligonucleotide binding (section “SSIs and the concept of restricted PS”).

It has been suggested that nucleoli assemble by PSCP combined with complex coacervation [2]. Nevertheless, in this example too, PSCP obviously depends both on active rRNA synthesis initiated by an SSI of Pol I with a specific site on rDNA and on PAR synthesis, which, as discussed above, is also driven by an SSI, which is necessary for PARP1s adopting its active conformation.

#### 6.1.3. SGs

SGs are inducible cytoplasmic condensates accumulating mRNA, RNA-binding proteins, and small ribosomal subunits. SG assembly takes place under stressful conditions and is triggered by a sudden increase in protein-free unfolded mRNA concentration in the cytoplasm owing to a translation blockage [130]. Numerous research articles indicate that SGs emerge through the LLPS of G3BP proteins (G3BP1 and its homolog G3BP2) [91,131,132], which involves G3BP interaction with RNA [130]. A detailed molecular mechanism of this RNA-mediated condensation is described in ref. [130]. It has been found that G3BP under unstressful conditions adopts an autoinhibitory conformation stabilized by electrostatic intramolecular interactions between the disordered E-rich tract and the positively charged RG-rich region. Unfolded mRNAs released from polysomes outcompete this autoinhibitory binding, thereby inducing a G3BP conformational switch to a multivalent state. G3BP with the released RG-rich region engages in cooperative protein–RNA interactions giving rise to networked G3BP–RNA condensates of low protein density [130]. Therefore, SG assembly is mediated by PSCP [2].

Of note, a map of PAR-associated complexes arising upon genotoxic stress has revealed a strong PAR association with many proteins participating in SG formation, including G3BP [133]. PAR noncovalently binds to the G3BP RG-rich domain, which possesses properties of a bona fide PAR-binding motif. This means that PAR may function similarly to RNA by releasing the autoinhibitory G3BP fold. The formation of G3BP foci triggered by DNA-damaging stress is PAR-dependent because their assembly is abrogated by PARP inhibition; moreover, it has been found that PAR can act as a modulator of G3BP nuclear export [133].

As evident from this case study, PSCP underlying SG formation is preceded by an SSI. Similar to the example of DNA and PARP, the SSI of unfolded RNA or PAR with a specific region of G3BP is necessary for the “PS on” conformational switch of this primary PS driver.

#### 6.1.4. Pathological Aggregates

Nucleic acids have a strong influence on the conformational changes and PS of amyloidogenic proteins [134]. PARP1 overexpression is associated with a list of central-nervous-system disorders related to abnormal protein aggregation [124,135]. Under physiological conditions, PAR regulates the formation and disassembly of SGs containing amyloid proteins (such as α-syn, amyloid β, tau, FUS, TDP-43, or hnRNPA1), whereas the dysregulation of PAR levels may cause amyloid aggregation [124]. For instance, PAR has been shown to trigger and accelerate the fibrillization of α-syn [123].

Recent results obtained by Rhine et al. suggest that PAR can trigger FUS condensation in a manner completely different from that of RNA. These investigators found that PAR chains longer than four monomers can alter FUS conformation into a LLPS-prone form via a transient interaction [136]. The FUS primed by a short-lived interaction with PAR can initiate the assembly of condensates, which persist even after PAR degradation by PARG [136]. Notably, the PAR-free fraction of FUS preincubated with PAR retains the ability to nucleate FUS condensation [136], transferring a seeding-competent conformation similarly to the “templated misfolding” mechanism [86]. Of particular importance is the observation that a PAR concentration sufficient for FUS PS (1 nM) appears to be manifold lower than $K_{d_{app}}$ (>200 nM) for PAR–FUS binding [136]. FUS aggregation induced by substoichiometric amounts of PAR has also been demonstrated by Altmeyer et al. [116]. In contrast to PAR, RNA associates with FUS stably ($K_{d_{app}}$∼5 nM) and is necessary for the persistence of FUS–RNA condensates because they completely dissolve upon RNase treatment [136].

This example, although shown only in vitro, is unique in that it is perhaps the only phenomenon where the persistence of the initial SSI is not required to maintain the phase equilibrium. Trace amounts of PAR are sufficient to generate the LLPS-prone self-reproducing FUS conformation, and PAR performs essentially a catalytic rather than structural function.

### 6.2. PAR and the Formation of Networks

#### 6.2.1. ASK3 Condensates

After osmolarity-induced swelling or shrinkage, the cell recovers its initial volume with the participation of apoptosis signal-regulating kinase 3 (ASK3), which bidirectionally switches its kinase activity under osmotic stress [137]. Protein phosphatase 6 (PP6) is a direct ASK3 phosphatase that interacts with ASK3 in an osmolarity-dependent manner and inactivates ASK3 via dephosphorylation [138]. The PP6–ASK3 interaction is a core stage in the cellular response to hypo- and hyperosmotic conditions [138].

Macromolecular crowding associated with hyperosmotic shock leads to the emergence of ASK3 condensates distinct from SGs, which also arise under extreme hyperosmotic conditions [120]. ASK3 condensates transduce an osmosensing signal into ASK3 inactivation [120]. These structures are formed by PS that has a “spinodal decomposition-like” pattern [120] hardly observed in vivo for any other phase-separated condensates. It is worth mentioning that although ASK3 inactivation is neither necessary nor sufficient for the ASK3 condensation, ASK3 condensation is required for its inactivation [120]. It is reported that the interaction between PP6 and ASK3 is enhanced under hyperosmotic conditions because PP6 and ASK3 condensates partly share a phase boundary, where this interaction and exchange of dephosphorylated and still phosphorylated ASK3 molecules proceeds [120]. It has been proposed that the PP6–ASK3 interface is a channel for PP6 and ASK3 movements within the crowded environment [120].

Even though ASK3 condensates emerging in vivo behave as liquid droplets, they are solid-like in vitro, judging by fluorescence recovery after photobleaching in both cases [120]. The addition of either a polyA oligoribonucleotide or PAR maintains the liquidity of ASK3 condensates in vitro. The cells deficient in PAR or those expressing an ASK3 mutant insensitive to PAR regulation form ASK3 condensates with significantly longer times of fluorescence recovery after photobleaching [120]. Consequently, PAR is not a necessary driver of the assembly of ASK3 condensates but serves as a factor regulating their viscoelasticity. It has been suggested that PAR functions as a multivalent intermediary that coordinates the transient network of interactions between ASK3 molecules [120]. Indeed, as demonstrated later, the liquid-like morphology is suppressed by tight interactions between protein units, and electrostatic shielding regulates the strength of ASK3–ASK3 binding [139]. Bulky PAR may also serve as a “loosening filler” increasing ASK3 movement within condensates and facilitating the interaction between ASK3 and PP6 around phase boundaries [120]. It has been found that PAR depletion reduces ASK3–PP6 interaction, whereas PARP1 overexpression inhibits ASK3 dephosphorylation under hyperosmotic stress [120].

It should be emphasized that ASK3 forms condensates only in response to hyperosmotic conditions, and PAR keeps ASK3 condensates in the liquid phase for ASK3 inactivation only under hypertonicity.

#### 6.2.2. Biomineralization

Bone biomineralization is a vitally important physiological process of depositioning apatitic calcium phosphate [140] within a proteinaceous extracellular matrix. In both cortical and trabecular bone, mineral platelets are highly organized around well-ordered collagen fibrils [122]. For a long time, it has been unclear how ${Ca}^{2+}$ is locally incorporated selectively over other metal ions and why collagen fibrils are preferred nucleation sites [122].

In 2014, by nuclear magnetic resonance spectroscopy, Chow et al. unexpectedly detected a large amount of PAR in a calcifying growth plate of a developing fetal bone [70]. Indeed, during osteogenic differentiation, osteoblasts produce hydrogen peroxide, which triggers PARP1 activation [141]. The overactivation of PARP1 depletes cellular NAD${}^{+}$ and ATP pools, thereby leading to cell death [142]. The massive necrosis of osteoblasts occurs in the zones of bone tissue mineralization, with 65% to 85% of bone osteoblasts suffering this fate [143]. Osteoblast necrosis used to be considered necessary for freeing space for the nascent mineral crystals. In contrast, because cell contents are released into the surrounding space during necrosis, the key purpose of osteoblast necrosis may actually be the delivery of PAR to the extracellular matrix of developing bone [70]. Dynamic light scattering assays point to the formation of amorphous PAR-${Ca}^{2+}$ spheres whose radii increase dramatically with ${CaCl}_{2}$ addition to the PAR solution. In contrast to ${Ca}^{2+}$, the emergence of such PAR structures is undetectable in the presence of other biologically relevant divalent metal ions ${Mg}^{2+}$ and ${Zn}^{2+}$, except for ${Mn}^{2+}$ (but the radius of ${Mn}^{2+}$-PAR spheres does not increase when ${Mn}^{2+}$ concentration is raised). When used as a control, DNA does not form a spheroidal structure with ${Ca}^{2+}$ [122].

**Figure 2:**
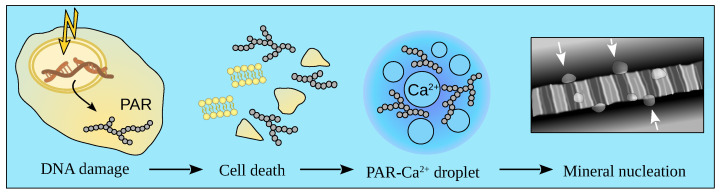
PAR and bone mineralization. Oxidative damage in differentiating osteoblasts triggers PARP1 overactivation inducing cellular necrosis and delivering PAR to the extracellular matrix. PAR forms dense liquid droplets with ${Ca}^{2+}$ ions that bind to the surface of collagen fibrils, thus initiating the nucleation of mineral platelets [122].

PAR-${Ca}^{2+}$ droplets condense on the surface of collagen fibrils, thus localizing to certain areas [122]. It was proposed in that publication that this highly organized pattern of calcium deposition is ensured by the electrostatic interaction between negatively charged PAR and the positively charged collagen C terminus [122] (see Figure 2). PARP1 inhibitors suppress bone mineralization in vitro and in vivo [122]. In a pathological state, the assembly of extracellular PAR-${Ca}^{2+}$ condensates is the cause of vascular calcification [122].

Extracellular PS has been described as protein-centric, whereas intracellular condensates are usually organized with the participation of RNA [97]. Therefore, the biomineralization process represents an unusual case of extracellular nucleic-acid-centric PS. As discussed in the section “Low complexity and polyA structure,” RNA in the presence of ${Mg}^{2+}$ forms a viscoelastic network [103]. Therefore, it can be expected that the complex coacervation of PAR with calcium ions also occurs along with the formation of a PAR intermolecular network, and the process of biomineralization can be considered PAR-driven PSCP.

## 7. Conclusions

The concept of PS as the leading force in the organization of living matter at various levels, from the cell [144] to the organism [122] and even beyond [145], has aroused great interest in the scientific community. The applicability of the basic LLPS model described by Flory–Huggins theory to real-world processes in vivo has been augmented by a strengthening of the theoretical basis [146], by means of more stringent criteria for evaluating experimental data [34], and finally, by attempts to link the LLPS concept with the classic structure–function paradigm [1,2].

Regardless of the perspective on macromolecular self-organization, its key participants are multivalent (LLPS) and capable of SSI (restricted PS) and of the formation of networks of interactions (PSCP) between proteins and nucleic acids.

In this review, we explored the role of PAR and the interactions mediated by this polymer in the assembly of various phase-separated condensates to understand how much SSIs and networking in these case studies help to build the final structure having properties of a separate phase.

At least four of the considered examples of PAR-containing condensates arise via restricted PS. Two of them (DNA repair foci and the nucleolus) require the maintenance of phase-initiating PS throughout their lifetime, which is determined functionally (the repair compartment is needed as long as DNA damage persists, and the nucleolus is necessary as long as there are rRNA transcripts for assembling ribosomal subunits).

In three of these examples, SSI is required for a conformational rearrangement of a protein to activate the “PS on” mode. In the case of DNA repair compartments, PS is triggered by local PAR synthesis, which is based on the SSI of PARP with damaged DNA. The “key” for the removal of autoinhibition of the enzyme is a DNA molecule containing a strand break, and the synthesized PAR performs a scaffold function in the organization of repair foci.

PAR itself can also act as the “key” by changing the conformation of LLPS drivers. In the case of SGs induced by a genotoxic insult, binding to PAR must prevent the autoinhibition of G3BP and its translocation into the cytoplasm. As for FUS compartments forming in vitro, an SSI of PAR and FUS alters FUS conformation into an LLPS-prone form possessing a capacity for prion-like spreading.

Aside from SSI, networking can contribute to the formation of these PAR-containing condensates, e.g., the networking taking place in the case of the nucleolus and SGs.

Nonetheless, two examples (ASK3 condensates and the biomineralization process) are closer to the general PS mode. When ASK3 condensates and biomineralization coacervates are assembled, it is already difficult to isolate a phase-initiating SSI. The former are characterized by maturation over time [120], which is inherent in PSCP-derived condensates [2], and a theoretical model [139] supports the hypothesis about the possible participation of PAR in network formation. According to the case studies examined here, only ASK3 condensates are completely independent from PARP activation after DNA damage because crowding serves as a trigger for their formation under hyperosmotic conditions. It is also noteworthy that during the emergence of ASK3 condensates, a spinodal decomposition-like pattern is observed, indicating the absence of specific nucleation sites. In the process of PAR-${Ca}^{2+}$ biomineralization, droplets are formed through complex coacervation; in this case, we only assume the formation of a network of interactions involving PAR by analogy with RNA.

It can be concluded that SSIs and networking, individually or together, are indeed important components of PS processes in vivo. Nevertheless, further efforts are necessary for clarifying the role and mechanism of PS in the cell.

## Data Availability

Not applicable.

## Abbreviations

The following abbreviations are used in this manuscript:

LLPS	liquid–liquid phase separation
PSCP	phase separation coupled to percolation
PS	phase separation
SSI	site-specific interaction
Ub	ubiquitin
SUMO	small ubiquitin-like modifier
PAR	poly(ADP-ribose)
IDR	intrinsically disordered protein region
RRM	RNA recognition motif
SG	stress granule
PTM	post-translational modification
FC	fibrillar center
DFC	the dense fibrillar component
GC	granular component
